# Combined Visco-Trab operation: A dual filtration pathway for management of advanced glaucoma—midterm results

**DOI:** 10.1007/s10792-021-01698-0

**Published:** 2021-02-06

**Authors:** Tarek M. Eid, Ezz El-Din M. Ibrahim, Ahmad Zaid

**Affiliations:** 1 Ophthalmology Department, Faculty of Medicine, Tanta University, Tanta, Egypt; 2 Glaucoma and Cataract Unit, Magrabi Hospitals and Centers, Cairo, Egypt; 3 EyeCity Center, New Cairo, Egypt; 4 Glaucoma and Cataract Unit, Magrabi Hospitals and Centers, Riyadh, Saudi Arabia; 5 Glaucoma and Cataract Unit, Magrabi Hospitals and Centers, Mascat, Oman

**Keywords:** Advanced glaucoma, Visco-trab operation, Dual filtration, Midterm results

## Abstract

**Purpose:**

To study midterm efficacy and safety of combined Visco-Trab operation for management of advanced glaucoma.

**Methods:**

168 eyes of 148 patients with advanced glaucoma had Visco-Trab operation (a merge of both viscocanalostomy and trabeculectomy operations). Mean follow-up was 29.1 ± 22.2 months. Criteria of success were intraocular pressure (IOP) of 14 mmHg or less with or without glaucoma medications, with no devastating complications, loss of light perception, or additional glaucoma surgery.

**Results:**

IOP, number of glaucoma drops, and visual field mean deviation were significantly reduced (11.9 ± 5.6 mmHg, 0.7 ± 1.2, and 14.2 ± 6.3 dB, compared to preoperative values of 24.4 ± 9.9 mmHg, 2.8 ± 1.4, and 17.3 ± 6.3 dB, respectively). Success was reported in 136 of 168 eyes (81%) without (100 eyes, 59.5%) or with (36 eyes, 21.5%) glaucoma medications. A functioning bleb was seen in 2/3rd of eyes; diffuse (59 eyes, 35%) and thin ischemic (54 eyes, 32%). Predictors for failure to achieve the target IOP included previous ocular (*p* = 0.01) or glaucoma (*p* = 0.04) surgery, number of preoperative glaucoma medications (*p* = 0.029), and severity of glaucoma (*p* = 0.058).

**Conclusion:**

Combined Visco-Trab operation proved safe and effective, on midterm follow-up, in reducing IOP to the proposed target level in eyes with severe glaucoma via enhancing internal and external filtration.

**Supplementary Information:**

The online version contains supplementary material available at (10.1007/s10792-021-01698-0).

## Introduction

Patients with advanced glaucoma (near total cupping of the optic disc and advanced visual field defect encroaching on fixation) are high-risk surgical candidates [1]. Complications of current surgical interventions, namely subscleral trabeculectomy with mitomycin C application (MMC-Trab), include high incidence of hypotony-related complications after the filtering operation or after suture cutting (flat anterior chamber, choroidal effusion or hemorrhage etc.) or postoperative pressure rise due to tight flap closure. This turbulence of postoperative IOP may endanger the compromised optic nerve and the residual vision[2].

Visco-Trab operation constitutes combining viscocanalostomy and trabeculectomy with Mitomycin C in a single technique in order to increase both internal and external flow of aqueous postoperatively [3]. Surgical technique included lamellar and deep scleral flap dissection, deroofing and viscodilation of Schlemm’s canal (SC), penetrating trabeculectomy, peripheral iridectomy, and tight flap closure. The early postoperative IOP reduction after Visco-Trab was mostly attributed to improved internal flow mechanisms, while external filtration is minimized by tight scleral flap closure. Subsequent scleral flap suture cutting, when needed, allows subconjunctival drainage of aqueous. Persistent IOP lowering is attributed partly to improved conventional outflow pathways and partly to external filtration. The controlled hypotony produced by enhancing internal filtration and limiting external filtration plays a major role in reducing IOP to the target level during the early postoperative period without pressure spikes or severe complications related to excessive filtration [3]. In a comparative study of the contralateral eye, comparing combined Visco-Trab operation to MMC-Trab proved a similar efficacy over a longer follow-up period with reduced postoperative complications [4].

In this study we evaluated the midterm efficacy and safety of Visco-Trab operation in terms of controlling IOP to a low and stable level with less eventful postoperative course.

## Patients and methods

A retrospective analysis of collected data for previous prospective studies was conducted. 168 eyes of 148 patients with advanced to end-stage glaucoma surgically treated with Visco-Trab operation in the Glaucoma and Cataract Unit at Magrabi Eye and Ear Center, Jeddah, Saudi Arabia, from 2006 till December 2013 were included in the analysis. A local institutional review board was obtained which meets the tenets of the Declaration of Helsinki, and an informed consent was signed by every patient. Advanced glaucoma in this study was defined as near total cupping of the optic disc, severe visual field defect encroaching on central 5 degrees of fixation in at least one quadrant or tubular field defect [1]. In some patients, reliable visual field cannot be obtained because of poor vision, dense cataract, or unreliability of the test. For these patients, glaucoma severity was judged by degree of damage of optic disc, IOP, and patient’s compliance. Surgical treatment for these patients was indicated when there was lack of proper control of IOP to the required target level with maximally tolerated medical therapy. The risk–benefit ratio of surgical management was explained to the patient, and a consent form was obtained. Patients with visually significant cataract had separate-site phacoemulsification combined with the glaucoma procedure.

## Surgical technique (Fig. 1)

**Fig. 1:**
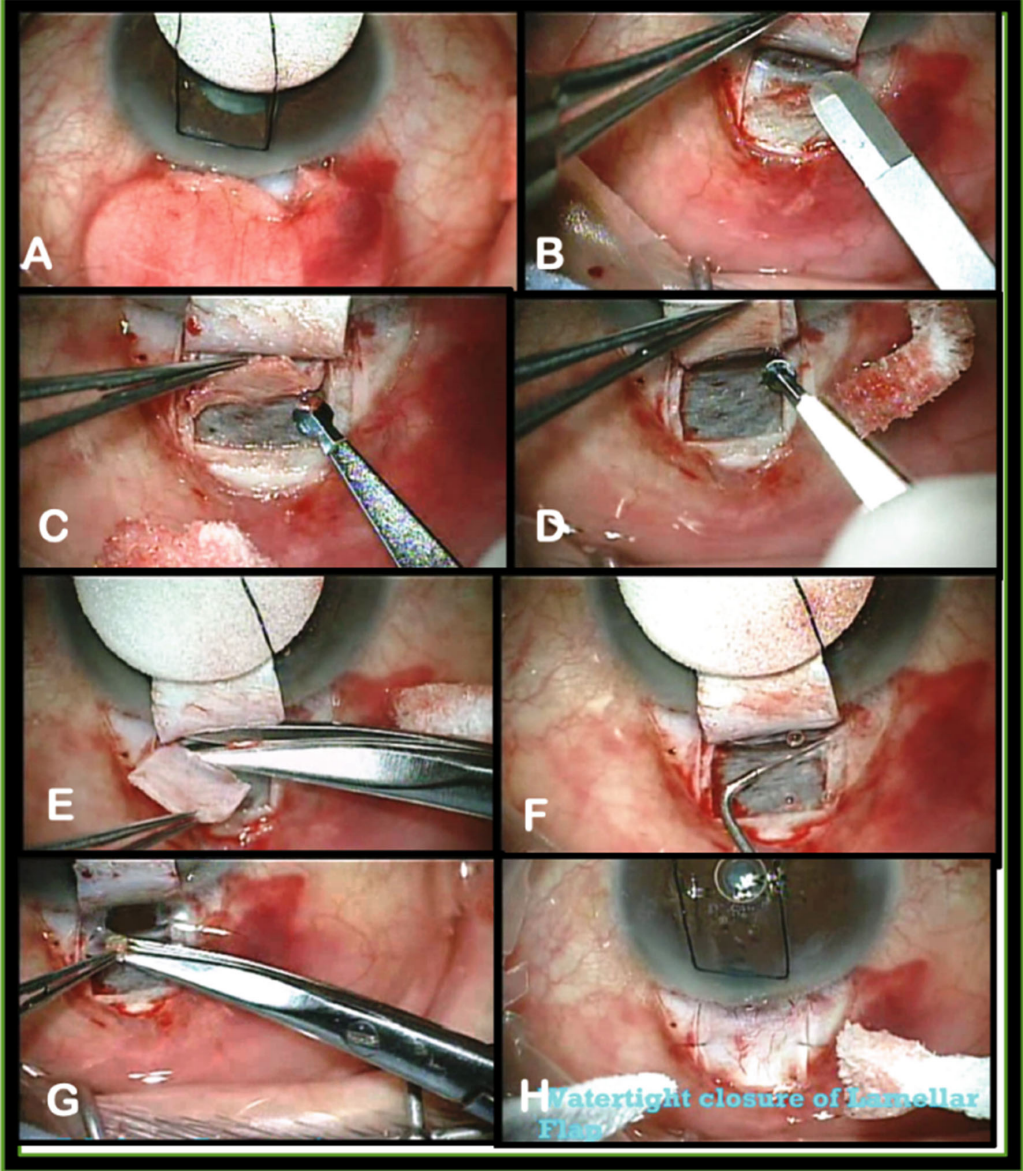
Steps of combined Visco-Trab operation for advanced glaucoma. a Fornix-based conjunctival flap and subconjunctival Mitomycin C 0.3 mg for 3 min. b A 4 × 4 mm lamellar scleral flap (1/3–1/2 of thickness) extending 1 mm in clear cornea. c Deep scleral flap dissection 0.5 mm inside edge, leaving thin scleral over choroid d Exposure & deroofing of Schlemm’s canal (No Descemet’s window exposure). e Excision of deep scleral flap creating a scleral lake. f Dilation of SC with sodium hyaluronate. g Penetrating trabeculectomy and peripheral iridectomy. h Water tight closure of lamellar scleral flap and conjunctival flap

The procedure was performed under topical or peribulbar anesthesia without application of Honan bulb. General anesthesia was given in young or uncooperative or sometimes in single-eyed patients. The globe is rolled down with superior corneal traction suture with 7/0 silk. The filtering site is located slightly nasal or temporal to the 12 o’clock position. Details of the procedure are explained in previous studies [3, 4]. After preparing the site of surgery and Mitomycin C application, a 4 × 4 mm rectangular lamellar sclera flap was outlined and dissected anteriorly to within 1 mm of clear cornea. A second deeper rectangular flap was outlined 0.5 mm inside the border of the first and dissected using a special dissecting sclera pocket knife (Grieshaber, Switzerland), leaving only a thin translucent layer overlying the choroid. Forward dissection in this plane continues until Schlemm’s canal (SC) is identified and deroofed. Dissection continues through the lumen and extends forward until it exceeds Schwalbe’s line and Descemet’s membrane is seen. No blunt dissection of Descemet’s window is performed as in routine viscocanalostomy. A specially designed viscocanalostomy cannula (Grieshaber, Switzerland), with an outer diameter of 165 µm was introduced into the ostia of SC, right and left, to inject sodium hyaluronate 1% (ProVisc, Alcon) into the canal. The cannula is introduced inside the canal for a short distance, and a small amount of viscoelastic is injected and repeated few times on each side. The trabeculo-Descemet’s membrane is incised right at the junction with the anterior border of the floor of SC, and a penetrating deep sclero-corneal block was excised for approximately 1.5 mm in length and 2 mm in width. A wide peripheral iridectomy is performed, and the lamellar sclera flap is closed watertight by 4 10/0 Nylon sutures, 2 at the distal corners and 2 on the vertical limbs. The AC is reformed, and the conjunctiva is closed with 2 corners and 2 transverse-mattress 10/0 Nylon sutures.

When the glaucoma procedure is combined with phacoemulsification, once Schlemm’s canal is deroofed, the surgeon switches his position to the temporal side to do the cataract operation through a clear-cornea incision. At the end of the phaco procedure, the AC is reformed and the corneal wound is secured by one 10/0 Nylon suture and the surgeon reverts back to upper position to complete the filtering operation.

Postoperatively patients used antibiotic-corticosteroid eye drops every 2 h for one week and then tapered gradually over 5 weeks. Cycloplegic-mydriatic eye drops were used for one week and may be continued if there were signs of early inflammation, shallow AC, hypotony, or anticipated risk of aqueous misdirection. Patients were examined at day 1 and 3 then at 1, 2, 4, and 8 weeks and after that periodically every 3 months. If the pressure was elevated, digital massage, focal compression, and LSL, needling with flap lift, or bleb revision with subconjunctival MMC injection were performed according to the IOP level and degree of filtration.

A minimal of 3 months follow-up was required to be included in the analysis. Criteria of Success were IOP of 14 mmHg or less at the last follow-up, with no devastating postoperative complications or loss of vision or additional glaucoma surgery.

Descriptive statistics of numeric (mean ± standard deviation) and categorical (count and percentage) variables for patients’ eyes, and glaucoma characteristics, operative data, and postoperative course were listed and tabulated. Paired t-test was used to compare IOP, number of glaucoma medications, visual field mean deviations, horizontal cup-disc ratio, and logMar visual acuity preoperatively and at the last follow-up evaluation. Kaplan–Meier survival analysis of probability of failure of Visco-Trab operation to achieve an IOP of 14 mmHg or less was plotted over the follow-up period. Predictors of failure to achieve the target pressure were studied using logistic regression analysis with a significance level of *p* ≤ 0.05.

## Results

One hundred ninety-seven eyes were operated with Visco-Trab operation throughout the study period, 25 of them had less than 3 months of follow-up in their records and did not match the inclusion criteria. Additional 4 eyes were converted to classic MMC-Trab and were excluded from the analysis.

Table 1 lists demographic data, characteristics of the study eye and type and severity of glaucoma. Seventy percent of the study patients were males (103 patients) and almost 60% of them (87 patients) were above 50 years of age. More than half of the eyes had primary open-angle glaucoma and 20% (34 eyes) had primary angle closure glaucoma. Three quarters of the eyes received previous glaucoma treatment, and two-thirds of them had a preoperative IOP of 25 mmHg or less. Table 2 describes operative data, intraoperative complications, early postoperative course, postoperative complications and additional surgical interventions. Self-absorbing hyphema was the most common early postoperative complications (21 eyes, 12.5%) followed by shallow to flat anterior chamber (19 eyes, 11.4%; 3 eyes required added maneuvers to reform the anterior chamber). Three eyes (1.8%) suffered from postoperative hypotony with choroidal effusion, two of them required choroidal tap to drain the fluid. Scleral flap lift with a 27-gauge needle on the slitlamp (14 eyes, 8.3%) or bleb revision with additional subconjunctival mitomycin C injection (11 eyes, 6.5%) were done in eyes after failure of laser suture lysis to augment external filtration.

**Table 1:** Patients’ and eyes’ characteristics (*n* = 168)

Variable		Frequency	Percentage
Sex (*n* = 148)	Male	103	69.6
Age group (n = 148)	> 50 years	87	58.8
Study eye	Right eye	81	48.2
Previous glaucoma treatment		125	74.4
Previous glaucoma surgery (MMC-Trab)		27	16.1
Preoperative visual acuity	> 20/40	93	55.4
	20/40–20/200	53	31.5
	< / = 20/400	22	13.1
Preoperative IOP (mmHg)	< / = 25	111	66.1
	> 25	57	33.9
Lens status	Clear	116	69.0
	Cataract	24	14.3
	Pseudophakic	28	16.7
Preoperative visual field changes	Double arcuate scotoma	6	3.6
	Hemifield defect	6	3.6
	Advanced defect not affecting fixation	17	10.1
	Advanced defect splitting fixation	64	38.1
	Tubular field defect	21	12.5
	Total field loss	12	7.2
	Not done	42	25.0
Glaucoma diagnosis	Primary open-angle	89	53.0
	Low-tension	4	2.4
	Primary angle closure	34	20.3
	Congenital	14	8.3
	Juvenile	8	4.8
	Angle recession	8	4.8
	Pseudoexfoliative	5	3.0
	Pigmentary	2	1.2
	Steroid-induced	3	1.8
	Uveitic	1	0.6
Glaucoma severity	Advanced	121	72.0
	End-stage	47	28.0

**Table 2:** Operative data and postoperative course of study eyes after Visco-Trab operation

Variable		Frequency	Percentage
Anesthesia	Topical	82	48.8
	Peribulbar	68	40.5
	General	18	10.7
Intraoperative complications			
	Vitreous loss from sclerostomy	2	1.2
	Descemet membrane detachment during viscodilation	3	1.8
	Choroidal exposure during deep flap dissection	4	2.4
IOP measurement within the first postoperative week			
	< / = 10 mmHg	133	79.2
	> 10 mmHg	35	20.8
Early postoperative complications		38	22.6
	Hyphema	21	12.5
	Shallow anterior chamber	16	9.6
	Flat anterior chamber	3	1.8
	Leaking conjunctival hole	2	1.2
	Choroidal effusion	3	1.8
	Suprachoroidal hemorrhage	1	0.6
Postoperative laser suture lysis			
	Within one week	4	2.4
	Between 1 and 4 weeks	36	21.4
	Between 1 and 3 months	5	3.0
Added postoperative surgical intervention			
	Anterior chamber reformation	3	1.8
	Repair of conjunctival hole Scleral flap lift on slitlamp	2 14	1.2 8.3
	Bleb revision and subconj 5-MMC	11	6.5
	Choroidal drainage	2	1.2
	Repair of Descemet membrane detachment	1	0.6
Cataract extraction during postoperative course		12	7.1

Mean follow-up was 29.1 ± 22.2 months (range 3–86). Mean pre- and postoperative IOP, number of glaucoma medications, visual acuity, and visual field mean deviation are demonstrated in Table 3. Mean IOP and number of glaucoma medications were reduced significantly at the last follow-up evaluation in comparison with their preoperative levels (11.9 versus 24.4 mmHg and 0.7 versus 2.8, respectively, *p* < *0.001*). On the other hand, the mean values of cup-disc ratio, visual field mean deviation, and logMar of visual acuity showed stable course over the follow-up length without any significant change from preoperative values.

**Table 3:** Mean, standard deviation, minimum, and maximum values of numeric variables preoperatively and at the last follow-up in the study

Variable		Mean	SD	Minimum	Maximum	*P* value
Age (years)		51.1	18.9	4	85	
Length of follow-up (months)		29.1	22.2	3	86	
IOP (mmHg)	Preoperative	24.4	9.9	10	55	< 0.001*
	At last follow-up	11.9	5.6	1	31	
Number of glaucoma drops	Before surgery	2.8	1.4	0	5	< 0.001*
	At last follow-up	0.7	1.2	0	4	
Cup/disc ratio (horizontal)	Before surgery	0.9	0.1	0.5	1.0	0.9
	At last follow-up	0.9	0.4	0	1.0	
Visual field mean deviation (dB)	Preoperative	17.3	6.7	6.2	32.4	0.2
	At last follow-up	14.2	6.3	4.5	24	
LogMar of visual acuity	Preoperative	0.5	0.3	0	1.0	0.9
	At last follow-up	0.5	0.3	0	1.0	

**Fig. 2:**
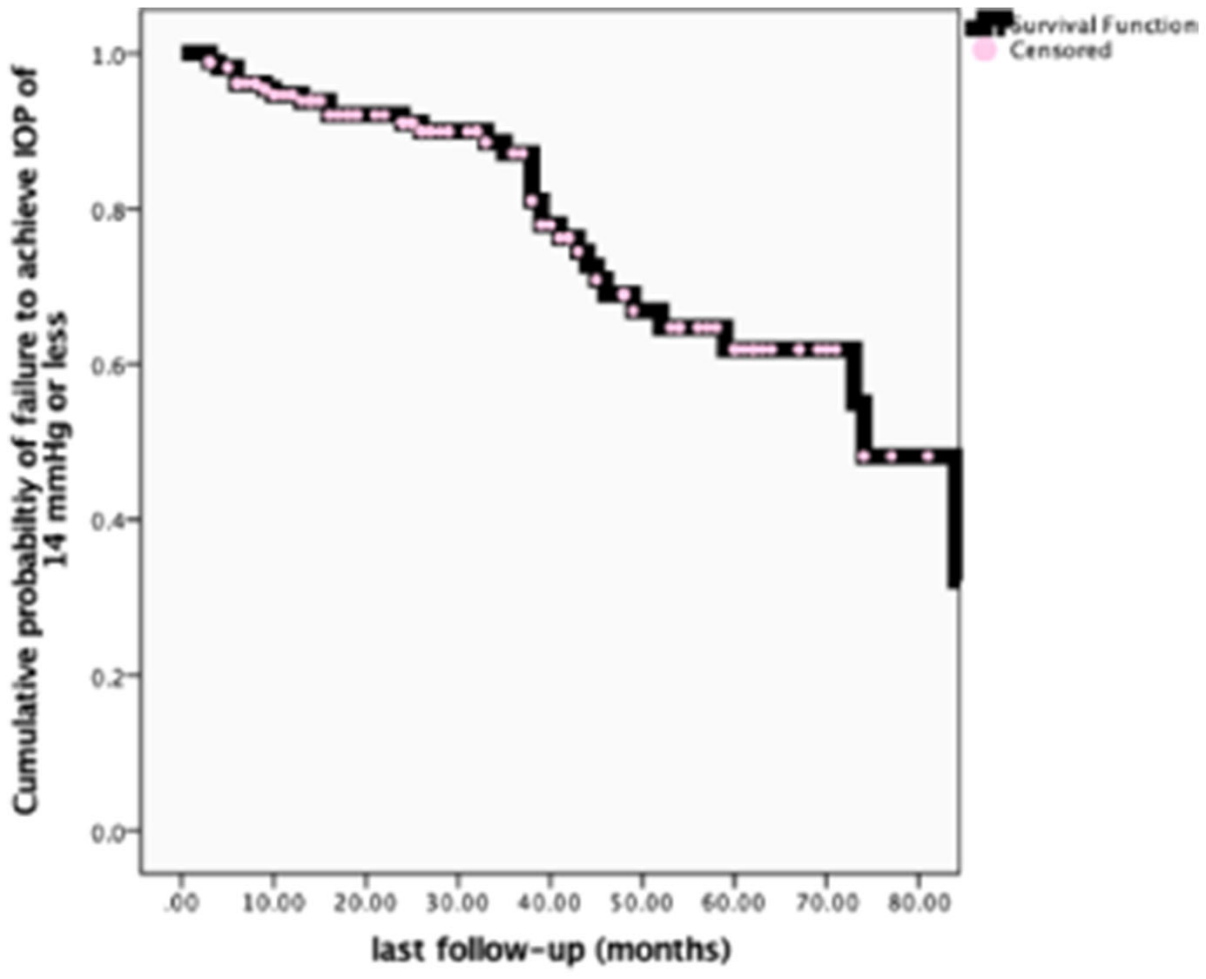
Kaplan–Meier survival analysis of probability of failure of Visco-Trab to achieve a pressure of 14 mmHg or less necessary for management of advanced glaucoma over the follow-up period

Table 4 presents the number and percentage of eyes that achieved an IOP of 21 mmHg or less with or without antiglaucoma medications, as well as the number and percentage of eyes that did or did not achieve the target pressure (≤ 14 mmHg) at last follow-up visit. Eighty one percent of eyes (136 of 168 eyes) achieved a target IOP of 14 mmHg or less without (100 eyes, 59.5%) or with glaucoma drops (36 eyes, 21.4%). This percentage of success (both complete and qualified) increased to 95.3% when a pressure of 21 mmHg or less was used as a cutoff value. The recorded appearance of the filtering bleb at last visit examination was counted and is presented in Table 4. Almost two third of the eyes had a functioning bleb either diffuse (59 eyes, 35%) or thin ischemic (54 eyes, 32%) blebs. Cumulative probability of failure of Visco-Trab operation to achieve an IOP of 14 mmHg or less over the extended follow-up period was shown in the Kaplan–Meier analysis chart (Fig. 2).

**Table 4:** Complete and qualified success of achieving a postoperative IOP of 21 mmHg and 14 mmHg and the appearance of the associated filtering bleb at last follow-up evaluation

Variable		Frequency	Percentage
Filtering bleb appearance at last follow-up			
	Flat bleb	23	13.7
	Localized bleb	28	16.6
	Diffuse bleb	59	35.1
	Thin ischemic bleb	54	32.1
	Cystic bleb	4	2.4
IOP < 21 mmHg at last follow-up			
	Without antiglaucoma drops	130	77.4
	With antiglaucoma drops	30	17.9
	IOP higher than 21 mmHg	8	4.8
IOP < / = 14 mmHg at last follow-up			
	Without antiglaucoma drops	100	59.5
	With antiglaucoma drops	36	21.4
	IOP higher than 14 mmHg	32	19.0

Predictors for failure to achieve the target IOP of 14 mmHg or less at last follow-up evaluation (by logistic regression analysis) included previous ocular (*p* = 0.01) or glaucoma (*p* = 0.04) surgery, number of preoperative antiglaucoma medications (*p* = 0.029), severity of glaucoma (*p* = 0.058). Nonsignificant predictors in the multivariate model included old age (*p* = 0.1) and high preoperative IOP (*p* = 0.7) (Table 5).

**Table 5:** Predictors of failure to achieve IOP of 14 mmHg or less (logistic regression analysis)

	Regression coefficient	*p* value	Odd’s ratio	95% Confidence Interval	
				Lower	Upper
Old age	0.831	0.114	2.295	0.818	6.435
Preoperative IOP (> 25 mmHg)	0.218	0.653	1.244	0.480	3.219
Number of preoperative glaucoma drops	1.014	0.029*	2.757	1.108	8.860
Previous glaucoma operation	1.089	0.040*	2.972	1.048	8.422
Previous ocular surgery	1.380	0.011*	3.974	1.373	11.499
Severity of glaucoma	0.936	0.058	2.550	0.970	6.702

* indicate significant *p* value

## Discussion

Combined Visco-Trab aims, in a single procedure, to lower IOP and lessen the early postoperative complications in patients with severe glaucoma. Surgeons familiar with trabeculectomy and nonpenetrating surgery will find no difficulty in performing Visco-Trab without any learning curve. In Visco-Trab, unlike viscocanalostomy, the technique is easier since dissection is not extending over Descemet’s membrane where most macro-perforations occur. [5–9].

The combined Visco-Trab operation reduces IOP by enhancing both internal and external filtration of aqueous fluid. The internal filtration is enhanced by several mechanisms; (1) deep scleral flap dissection and excision creates a scleral lake that accommodates and directs aqueous to orifices of SC or to suprachoroidal space via the remaining thin scleral sheet; (2) dilation of SC with viscoelastic results in circumferential expansion of the canal and collector channels and focal ruptures of the inner wall of SC and juxta canalicular meshwork; (3) tight closure of the superficial lamellar scleral flap limits external flow to the subconjunctival space and directs all aqueous into conventional outflow pathways. On the other hand, subconjunctival flow is guaranteed over the long term via; (1) excision of a penetrating deep sclero-keratectomy (trabeculectomy) creates a guarded external fistula; (2) use of subconjunctival mitomycin C as an antifibrotic agent to reduce episcleral fibrosis; (3) delayed laser cutting of the sutures of the lamellar flap to augment external filtration when needed.

In this study, Visco-Trab operation for management of advanced glaucoma proved midterm efficacy in reducing IOP to 14 mmHg or less in 60% (complete success) and 81% (qualified success) of the eyes at the last follow-up. The total success rate increased to 95.3% when a pressure of 21 mmHg or less was used as a cutoff value. Our results were comparable to those of King and Stead [10]. They reported a success rate of 85% for achieving an IOP of 16 mmHg or less at year 5 follow-up after MMC-Trab for patients with advanced glaucoma. However, Law and associates [11], comparing initial versus repeat MMC-Trab for open-angle glaucoma, reported lower success rate in achieving an IOP of 15 mmHg or less (61% and 41%, respectively). On studying the outcome of viscocanalostomy in patients with advanced glaucoma at 1, 2, and 3 years follow-up by Tsagkataki and associates [12], complete success was 45%, 28%, and 31%, whereas qualified success was 67%, 66%, and 60%, respectively. On the other hand, combination of trabeculectomy with deep sclerectomy had an 83% complete success and 100% qualified success to achieve a 22 mmHg or less IOP after 12 months of follow-up [13]. The success rate was much less in a recent study by Sangtam and associates [14] using combined modified deep sclerectomy and trabeculectomy for various types of glaucoma at different stages of severity. Complete success in their study was 50% (for IOP < 22 mmHg) and 36% (for IOP < 16 mmHg), while qualified success was 70% and 47%, respectively. The difficulty to compare different studies in terms of success rates is due to variability in selection criteria of patient type, glaucoma diagnosis, disease severity; previous ocular surgery; primary versus repeat glaucoma operation; variable success criteria; and follow-up duration. In our Visco-Trab study, we had patients with various ethnicities, different types of glaucomas, and nearly one third of the eyes had previous ocular surgery, but all patients were categorized as advanced disease, operated by one surgeon, all had the same surgical technique, follow-up regimen, and criteria of assessment.

In the meantime, Visco-Trab operation safely reduced postoperative pressure spikes as well as hypotony-related complications that might result from excessive external filtration. Reported intraoperative complications were very few (Table 2) denoting that the procedure can be mastered easily. Micro- or macro-perforations are the most common complications of viscocanalostomy that occur during dissection of the descemet’s window, a step not required in Visco-Trab operation. Postoperative complications that required added surgical intervention included flat anterior chamber (3 eyes), choroidal effusion (3 eyes, 2 of them had choroidal tap). None of the eyes lost light perception or had other devastating complications (endophthalmitis, or atrophia). In a previous study [3], early postoperative complications were comparable in number; however, some complications were greater in severity after MMC-Trab than after Visco-Trab, such as anterior chamber shallowness (no eyes with flat chamber in the Visco-Trab group compared to 5 eyes in the MMC-Trab group), choroidal effusion (none required surgical drainage after Visco-Trab compared to 2 eyes after MMC-Trab), and surgically induced astigmatism. Additionally, patients required less postoperative surgical intervention in the Visco-Trab group (3 interventions compared to 8 in the MMC-Trab group). In another contralateral eye-controlled study [4], early postoperative complications were greater and postoperative surgical interventions were more frequent after MMC-Trab than after Visco-Trab operation. In a previous study on 60 eyes with advanced glaucoma having MMC-Trab, a 35% postoperative complication rate was reported (25% of the eyes had excessive filtration and shallow or flat anterior chamber and 15% had choroidal effusion). [15] A similar rate of postoperative complications after Trab with antifibrotic agents was reported in other studies. [16–19].

Several studies reported post-laser suture lysis complication rates between 21 and 42% after MMC-Trab [16, 20]. Viscoelastic dilatation of Schlemm's canal in Visco-Trab was shown to enhance internal filtration early postoperatively. This made suture cutting seldom necessary in the first two weeks after surgery despite tight closure of lamellar sclera flap. This relatively long interval may limit excessive flow of aqueous into the subconjunctival space with less adverse events. Morinelli and associates reported that a longer time interval between surgery and suture lysis may result in both a lesser degree of IOP reduction and a lower incidence of subsequent hypotony [20]. Delayed suture cutting postoperatively after Visco-Trab might be associated with early onset episcleral and scleral fibrosis. This, in some patients, may explain the lack of IOP lowering after laser suture lysis and necessitates invasive maneuvers such as needling and flap lift on the slitlamp (14 eyes) or bleb revision with subconjunctival mitomycin C in the operating room (11 eyes).

Most of the eyes had stable visual acuity and visual fields compared to preoperative level (Table 3). None of the eyes had “wipe-out phenomenon” after Visco-Trab neither in this study nor in our previous report [3, 4]. Costa and associates [21] reported that the risk of unexplained postoperative loss of central visual field does exist but is lower than 1% and is more likely to occur in older patients with macular splitting in the preoperative visual field. In another study, Topouzis et al. [22] reported no occurrences of “wipe-out” phenomenon in their series of end-stage glaucoma after MMC-Trab. They concluded that this sudden, unexplained postoperative loss of central vision is, at most, a rare complication and early surgical intervention should be considered in these patients. Stability of the visual filed mean deviation, cup/disc ratio, and visual acuity over the extended follow-up period (Table 3) indicates the importance of a low and stable target IOP for those glaucoma patients with advanced neuronal damage [23, 24].

Combined Visco-Trab, unlike nonpenetrating surgeries, can be performed for all types of glaucoma regardless of the angle appearance. Disadvantages of this technique include added surgical difficulty and time consumption in deep scleral block dissection & Schlemm’s canal exposure, extra cost of viscoelastic, a special knife and cannula not routinely used with MMC-Trab. In this study, sodium hyaluronate 1% is used instead of the high molecular weight Healon GV or Healon 5 which may have affected our results [25]. Tanito and coworkers [26] reported a comparable success rate using Healon 1% (Pharmacia, Japan), but they assumed that it may be related to the high incidence of postoperative hyphema.

Two third of eyes had functioning blebs either diffuse (59 eyes, 35%) or thin ischemic (54 eyes, 32%) which may be matching with almost 60% of eyes that achieved complete success. On the other hand, only a quarter of the eyes (45, 26.8%) required postoperative laser suture lysis despite the surgical plan of tight closure of the lamellar scleral flap in Visco-Trab. This significant long-term IOP reduction without suture cutting may be attributed partly to viscoelastic dilation of Schlemm’s canal and widening of trabecular pores which may provide long-lasting enhancement of internal filtration. In the meantime, even though the scleral flap is being closed watertight, there could be percolation of aqueous into the subconjunctival space assisted by blinking, ocular pulsation, digital massage, and reduced healing activity induced by antifibrotic agents (mitomycin C and topical steroids). Many of these patients develop a functioning bleb without the need for postoperative suture cutting.

Significant predictors for failure of Visco-Trab operation to reduce IOP to 14 mmHg or less at last follow-up evaluation included previous ocular surgery (*p* = 0.01), previous glaucoma surgical intervention (*p* = 0.04) surgery, and number of preoperative antiglaucoma medications (*p* = 0.029). Severity of glaucoma (advanced versus end stage) had border line significance (*p* = 0.058), whereas old age (*p* = 0.1) and high preoperative IOP (*p* = 0.7) were not significant predictors for failure to maximally reduce IOP after Visco-Trab technique. Law and coworkers^11^ reported that younger age and requirement for laser suture lysis were significant risk factors for failures in eyes with repeated MMC-Trab. Song and associates [27] found increased age, greater baseline IOP, limbus-based conjunctival flaps, and MMC duration > 1 min were associated with decreased risk of surgical failure of MMC-Trab for eyes with primary angle-closure glaucoma.

In conclusion, combined Visco-Trab operation proved safe and effective, on midterm follow-up, in reducing IOP to the proposed target level in eyes with severe glaucoma via enhancing internal and external filtration.

## Supplementary Information

This video presents Visco-Trab operation combined with clear cornea temporal phacoemulsification and in-the-bag foldable intraocular lens implantation in a patient with advanced primary open-angle glaucoma and posterior subcapsular cataract.Steps of combined Visco-Trab operation include fornix-based conjunctival flap and subconjunctival Mitomycin C 0.3 mg for 3 min followed by through wash with BSS. A 4x4mm lamellar scleral flap (1/3-1/2 of thickness) extending 1mm in clear cornea. Deep scleral flap dissection 0.5 mm inside edge, leaving thin scleral over choroid. Exposure & deroofing of Schlemm’s canal. When the glaucoma procedure is combined with phacoemulsification, once the canal is deroofed, the surgeon moves to the temporal side to do the cataract operation through a clear-cornea incision. At the end of the phaco procedure, the AC is reformed and the corneal wound is secured by one 10/0 Nylon suture and the surgeon reverts back to upper position to complete the filtering operation. Excision of deep scleral flap creating a scleral lake. Dilation of SC on either side with sodium hyaluronate. Penetrating trabeculectomy (1x2 mm) and wide peripheral iridectomy were done followed by water tight closure of lamellar scleral flap and conjunctival flap (mp4 98379 kb)

## Data Availability

All data available in data collection sheets and in SPSS files.
